# Dropout in a Longitudinal Survey of Amazon Mechanical Turk Workers With Low Back Pain: Observational Study

**DOI:** 10.2196/58771

**Published:** 2024-11-11

**Authors:** Nabeel Qureshi, Ron D Hays, Patricia M Herman

**Affiliations:** 1 RAND Health Care RAND Corporation Santa Monica, CA United States; 2 Division of General Internal Medicine and Health Services Research Department of Medicine University of California, Los Angeles Los Angeles, CA United States

**Keywords:** chronic low back pain, Mechanical Turk, MTurk, survey attrition, survey weights, Amazon, occupational health, manual labor

## Abstract

**Background:**

Surveys of internet panels such as Amazon’s Mechanical Turk (MTurk) are common in health research. Nonresponse in longitudinal studies can limit inferences about change over time.

**Objective:**

This study aimed to (1) describe the patterns of survey responses and nonresponse among MTurk members with back pain, (2) identify factors associated with survey response over time, (3) assess the impact of nonresponse on sample characteristics, and (4) assess how well inverse probability weighting can account for differences in sample composition.

**Methods:**

We surveyed adult MTurk workers who identified as having back pain. We report participation trends over 3 survey waves and use stepwise logistic regression to identify factors related to survey participation in successive waves.

**Results:**

A total of 1678 adults participated in wave 1. Of those, 983 (59%) participated in wave 2 and 703 (42%) in wave 3. Participants who did not drop out took less time to complete previous surveys (30 min vs 35 min in wave 1, *P*<.001; 24 min vs 26 min in wave 2, *P*=.02) and reported having fewer health conditions (5.88 vs 6.6, *P*<.001). In multivariate models predicting responding at wave 2, lower odds of participation were associated with more time to complete the baseline survey (odds ratio [OR] 0.98, 95% CI 0.97-0.99), being Hispanic (compared with non-Hispanic, OR 0.69, 95% CI 0.49-0.96), having a bachelor’s degree as their terminal degree (compared with all other levels of education, OR 0.58, 95% CI 0.46-0.73), having more pain interference and intensity (OR 0.75, 95% CI 0.64-0.89), and having more health conditions. In contrast, older respondents (older than 45 years age compared with 18-24 years age) were more likely to respond to the wave 2 survey (OR 2.63 and 3.79, respectively) and those whose marital status was divorced (OR 1.81) and separated (OR 1.77) were also more likely to respond to the wave 2 survey. Weighted analysis showed slight differences in sample demographics and conditions and larger differences in pain assessments, particularly for those who responded to wave 2.

**Conclusions:**

Longitudinal studies on MTurk have large, differential dropouts between waves. This study provided information about the individuals more likely to drop out over time, which can help researchers prepare for future surveys.

## Introduction

### Background

Conducting surveys on platforms such as Amazon Mechanical Turk (MTurk) have proliferated as a cost-effective and fast way of collecting data about health [1-3]. The number of studies using MTurk for social science research has been steadily increasing due in part to the ease of use, existing tools to support research activities, and quick turnaround for data collection [4]. In addition to the relatively low costs of conducting survey research with MTurk [5], another potential benefit is being able to reach participants and retain them in longitudinal studies [6]. If the goals of a research study involve having a representative sample of participants, it is important to assess how well MTurk can meet that need*.*

MTurk is one of many ways to collect nonprobability survey samples that are defined and created by researchers from a pool of available participants [7,8]. Previous research has found differences between the characteristics of MTurk respondents and the US general population. MTurk participants are generally younger, more likely to be female, White, have lower income, and have higher education levels compared with the US general population, differences that have persisted over time [9-14].

### Previous Work

Collecting a nonprobability versus a probability-based sample may depend on the research question. While “statistical sampling theory suggests that any estimate of a parameter will be more accurate when that parameter is estimated using data from a random sample” [7], adjustment approaches after sample collection may improve the comparability of a nonprobability sample to the general population [15]. However, nonresponse bias due to attrition in samples can significantly impact inferences drawn from either a probability or a nonprobability panel [16,17]. Attrition over time can reduce sample size, which lowers the power of any statistical analysis, while differential attrition can bias inference in less predictable ways [7]. Several methods exist to control for bias introduced by nonresponse, including sample weighting, that reduce the impact of nonresponse on inferences. Survey attrition has been noted as a critical concern with using MTurk [4]. Still, there is limited information about the effects of survey attrition in longitudinal studies using MTurk and the extent to which it limits the inferences that can be drawn [18].

Previous research has shown that nonresponse patterns vary by survey population and survey type in MTurk. In a 3-wave longitudinal study fielded from April 2020 to March 2021, MTurk respondents who were younger, Hispanic, and had self-rated difficulty with the survey were more likely to drop out in subsequent survey waves [19]. Rates of nonresponse for short-term studies (ie, a few days to a few weeks) tend to be lower than for long-term studies (ie, a month or more) [20]. Factors related to nonresponse vary by survey type, time between survey waves, and the underlying population [21-23]. Surveys that are longer and with greater response burden produce higher rates of nonresponse [24] in all types of longitudinal surveys [25,26], including internet survey panels [22,23]. Most of these studies have focused primarily on samples of the general population [19] rather than on subgroups with clinical conditions.

We use the Mercer et al [27] framework to assess the impact of nonresponse on estimation and bias in a longitudinal study of individuals with back pain. The authors propose a 3-element assessment to assess the impact of selection bias in survey estimates. We adapt this framework to and evaluate nonresponse, assuming the baseline data reflect the population and that nonresponse bias is similar to selection bias when assessing longitudinal surveys. The 3 elements proposed by Mercer et al [27] include “exchangeability” (whether all confounding variables are known and measured), “positivity” (whether the sample includes all necessary kinds of units in the target population), and “composition” (does the sample distribution match the target population concerning confounders, or can it be adjusted to match the target population). Assessing and addressing issues with exchangeability, positivity, and composition have been shown to improve inference in causal analysis and survey analysis to deal with selection bias issues. In this article, we use the same framework to improve inference from nonresponse bias in MTurk studies.

It is essential to understand the factors associated with attrition in longitudinal studies with internet panels, given their widespread use. To improve exchangeability, it is also important to understand and assess what factors could confound inference due to nonresponse. While studies have previously examined these issues among general populations, the factors associated with attrition may vary among populations with different health conditions. It is estimated that 39% of the US adult population has back pain [28]; back pain accounts for the largest share of years lived with disability in the United States [29]. Healthier individuals are more likely to respond to surveys, and longitudinal surveys risk losing an increasing number of less healthy participants in successive survey waves [30,31]. Given that health and pain are multidimensional, multiple measures of health and pain may be necessary to capture the confounding due to poorer health and increased pain. As more studies use surveys to assess back pain, nonresponse due to poorer health can significantly impact inference drawn from analyses, even longitudinal analyses, if differential attrition by pain status is observed. In addition, MTurk workers are known to have a high turnover rate [32]. The inability to follow up could be another important source of attrition.

### Goals of the Study

As a part of a more extensive study, we collected survey data on MTurk from individuals who self-identify as having back pain. To improve sample quality, we implemented a range of tactics to screen out poor-quality data, requiring that participants had completed several previous tasks and met an approval threshold, as well as postsurvey data cleaning to screen out those who reported having one or both of 2 fake health conditions included on the survey. What was left was a sample of self-selected, higher-quality participants who were surveyed 3 times over 6 months.

Because of the prevalence of individuals with back pain, attention to them, and the use of survey methods to assess their back pain, we analyzed data from a 6-month 3-wave longitudinal panel survey to (1) describe the patterns of survey responses and nonresponse among MTurk members with back pain, (2) identify factors associated with survey response over time (to assess “exchangeability”), (3) assess the impact of nonresponse on sample characteristics (to assess “positivity”), and (4) assess how well inverse probability weighting can account for differences in sample composition (to assess “composition”). We hypothesize that those with poorer health, more pain symptoms or severity, specific pain, and nonchronic pain will be least likely to respond to follow-up surveys. Weighting may be able to adjust to correct for nonresponse, but whether the sample is sufficiently varied is unclear.

## Methods

### Recruitment

We developed web-based surveys to collect data from MTurk participants and used the platform CloudResearch (formerly TurkPrime; Amazon) to field the survey in 2021 [33]. Individuals who reported having back pain at baseline (wave 1) were provided the opportunity to complete follow-up surveys after 3 months (wave 2) and 6 months (wave 3). We did not note in the wave 1 survey instructions that this was a longitudinal study because only those who met the inclusion criteria for the longitudinal study were asked if they wanted to participate in follow-up surveys. At the beginning of wave 2 and wave 3 recruitment, all eligible participants who consented to participate in follow-up survey waves were sent a recruitment email telling them the follow-up survey was available, that it would take approximately 25 minutes to complete, the payment for completing it, and that they had up to 5 weeks to return it. Weekly reminder emails (1-4 weeks after the recruitment email) were sent to all nonparticipants reiterating the timeline for survey completion, the approximate time to complete it, and the payment for completion.

Based on previous data collection efforts, we recruited individuals to have a final wave 1 sample of about 1500 individuals with back pain [9]. Those invited to participate at baseline had to have completed a minimum of 500 previous human intelligence tasks (HITs) on MTurk with a successful completion rate of at least 95%. No additional requirements were given to participate in the wave 1 survey. These threshold values were selected to enhance data quality. Previous research [34] and pilot tests of the survey found a 95% approval threshold and at least 500 completed HITs improve data quality and that limiting samples to ensure data quality does not limit the pool of available workers enough to restrict the sample to below the 1500-participant target [35]. While more recent studies [36] have shown that the approval rate is insufficient to ensure high-quality responses, we used a range of steps to ensure high-quality responses, including reputation, number of previous tasks, and attention checks (described in the Measures section). Given the structure of the MTurk interface, we are unable to determine the impact of the approval and completed HIT thresholds have on the sample profile (ie, we cannot quantify the number of individuals who tried to complete the survey but could not because of the thresholds for participation, as those individuals would not see the survey). Additional detail on data collection of wave 1 data is described by Qureshi et al [13].

### Ethical Considerations

All participants provided electronic consent at the beginning of the survey. Those who completed general health and back pain surveys at wave 1 were offered US $3.50 for their participation. Participants were offered an additional US $5 per subsequent completed survey (wave 2 and wave 3). All baseline participants (even those who did not participate in wave 2) were asked to participate in wave 3. Data were deidentified and are stored online [37]. All procedures were reviewed and approved by the research team’s institutional review board (RAND Human Subjects Research Committee FWA00003425; IRB00000051) and conforming to the Declaration of Helsinki principles. The study was funded by the National Institutes of Health or the National Center for Complementary and Integrative Health (Grant 1R01AT010402).

### Measures

The main outcome variable was participation in wave 2 and waves 2 and 3, defined as a binary outcome (0 if no participation and 1 if participation). We used several exposure variables, including self-reported demographic variables, self-reported health conditions, and self-reported back pain assessments.

Each survey asked about demographic characteristics (age, sex, race or ethnicity, employment status, income, education, and marital status) and health conditions. Health conditions were assessed in 2 forms. First, we asked “Have you EVER been told by a doctor or other health professional that you had…” for each of the following conditions: hypertension, high cholesterol, heart disease, angina, heart attack, stroke, asthma, cancer, diabetes, chronic obstructive pulmonary disease, arthritis, anxiety disorder, and depression. Then, we asked “Do you currently have…” for each of the following conditions: allergies or sinus trouble, back pain, sciatica, neck pain, trouble seeing, dermatitis, stomach trouble, trouble hearing, and trouble sleeping. We included these various measures of health to allow for the examination across various dimensions of health to support “exchangeability” for inference.

We also included 2 fake conditions in the survey that were used to screen out low-quality respondents. Individuals who endorsed one or both fake conditions were not asked to participate in the back pain follow-up survey if they endorsed having back pain. Overall, 15% (996/6832) of respondents endorsed one of these fake conditions, and their responses were believed to be dishonest or careless. Those reporting fake conditions were more likely to identify as male, non-White, to be younger, report more health conditions, and take longer to complete the survey. Their responses had less internal consistency reliability on several health measures than those who did not endorse a fake condition (Hays et al [38]).

Those who reported having back pain were asked to participate in a follow-up survey that included additional questions related to their back pain. If an individual opted not to continue, they would be paid for completing the first part of the survey and were not included in further analysis. The survey included questions about whether the respondent’s back pain was “chronic” according to 1 of 4 definitions (either that their back pain persisted for least 3 months, that their back pain persisted for at least 3 months, and they had pain at least half the days in the past 6 months, that a health provider told them that their pain is chronic, or that they believe their back pain is chronic). We also asked whether their back pain was due to a “specific” medical condition. We categorized individuals with back pain into 4 groups [39]—those with specific chronic back pain, those with specific nonchronic back pain, those with nonspecific chronic back pain, and those with nonspecific nonchronic pain. The survey also included the Impact Stratification Score (ISS) [40], Oswestry Disability Index (ODI) [41], Roland Morris Disability Questionnaire (RMDQ) [42], the Pain, Enjoyment of Life and General Activity scale (PEG) [43], and the Keele STarT Back Screening Tool (SBST) [44].

### Statistical Analysis

We report response rates to the wave 2 and 3 surveys among those responding to the wave 1 survey to assess the “positivity” of samples for inference. In addition, we report descriptive statistics on age, sex, race or ethnicity, income, education, marital status, self-reported health conditions, the proportion who endorsed back pain types, and back pain measure scores for those who participated in each survey wave. We report differences for those who did and did not complete the wave 2 survey and both the wave 2 and wave 3 surveys using *t* tests for continuous and chi-square tests for categorical variables.

Next, we report estimates from stepwise logistic regression models predicting response to wave 2 (model 1) and both waves 2 and 3 (model 2). We used a backward elimination with a selection criterion of α=.157 and a forward selection criterion of α=.05 to select the variables to include in the models [45]. These selection criteria determine whether a variable is included in the final model. Using a backward elimination with a selection criterion of α=.157 rather than α=.05 is meant to reduce overfitting of the final model, a common issue associated with stepwise models [46]. We report the odds of completing the subsequent surveys. Based on previous studies, age, sex, race, and ethnicity were included in the regression models [19,47]. We also examined education, marital status, income categories, employment, health conditions, type of pain, pain impact, and time to complete the questionnaire as predictor variables.

Finally, we used inverse probability score weighting to examine sample characteristics in waves 2 and 3 based on model 1 and model 2 results to assess how well the sample weights correct for nonresponse from in later waves to assess “composition.” Model weights are derived from estimated probabilities of completion using the aforementioned stepwise logistic regression models. By using inverse probability weights, we overweight respondents like those who drop out, approximating how the original sample would have looked if everyone responded to both follow-up waves. We included all candidate variables without backward elimination as a sensitivity analysis to derive inverse probability weights. Similar baseline characteristics between the full sample at baseline and weighted estimates for those who participated in later waves is an indication that observed variables can account for the level of bias introduced by sample attrition. All analyses were conducted using Stata software version MP17 (StataCorp) [48]. The study confirms to the STROBE (Strengthening the Reporting of Observational Studies in Epidemiology) checklist for cohort studies (Table S1 in Multimedia Appendix 1).

## Results

### Sample Characteristics

A total of 1678 adults who responded in wave 1 qualified to take subsequent surveys, that is, did not endorse a fake condition on the wave 1 survey and consented to participate in a future survey [13]. Of those who qualified to participate from the total sample in wave 1, 983 (59%) responded in wave 2. Of the 983 who responded in wave 2, a total of 703 (42% of wave 1 respondents) also responded in wave 3. The 8 respondents who only responded in waves 1 and 3 (ie, not in wave 2) were excluded from further analyses. Compared with those who did not respond, respondents in wave 2 were older, with higher income, more likely to never have been married, less likely to be Hispanic, less educated, and less likely to be employed full-time. We saw similar trends for those who responded in both waves 2 and 3 versus those who did not (Table 1).

Table 2 shows the overall sample distribution at wave 1 and response rates in waves 2 and 3. Generally, those who were older, those who were female, non-Hispanics, not married or living with a partner, and those at low (ie, US $0-US $39,999) and high income (more than US $60,000) were more likely to respond during waves 2 and 3 than their counterparts. These differences were more apparent when comparing wave 1 with wave 3. However, when comparing wave 3 response among those who responded in wave 2, response rates were generally 65%-75% and not systematically different by characteristic. In addition, the sample prevalence of health conditions was similar between the unweighted samples of those who participated in wave 2 only and those who participated in waves 2 and 3 (Table 2).

Those who responded in wave 2 completed the wave 1 survey in less time than those who did not respond in wave 2 (30 min vs 35 min, *P*<.001). Those who responded to the wave 3 survey also reported less time completing the wave 2 survey than those who did not respond (24 min vs 26 min, *P*=.02), similar to the time advertised to complete the survey. We found no differences in the time of day (morning, afternoon, evening, or nighttime) when the baseline survey was completed between the responders and nonresponders to the wave 2 survey and the waves 2 and 3 surveys.

Respondents in wave 2 had fewer reported health conditions than those who did not respond (5.8 vs 6.6, *P*<.001). A similar trend was observed for those who responded versus those who did not respond to both waves 2 and 3, though the effect was not significant (5.7 vs 6.0, *P*=.08). There were differences between responders and nonresponders in wave 2 for 15 conditions, with nearly all being less common for responders than nonresponders, except for arthritis, anxiety, and allergies. There were also differences between responders and nonresponders in waves 2 and 3, but for fewer (11) conditions (Table 3).

Respondents to wave 2 were less likely to have nonspecific low back pain and more likely to have chronic low back pain than those who did not respond, with similar patterns for those who did and did not respond to both the waves 2 and 3 surveys. Participants in wave 2 and in both waves 2 and 3 reported less pain intensity and pain interference, and better health on the ISS, ODI, RMDQ, PEG, and SBST measures (Table 4).

**Table 1:** Characteristics of those participating in wave 1 only versus those who also responded in wave 2 (at 3 months) and in waves 2 and 3 (at both 3 and 6 months).

Characteristic		Responded in wave 1 only (N=695), n (%)	Responded in wave 1 and 2 only (N=983), n (%)	*P* value (wave 1 vs wave 1 and 2)		Responded in all 3 waves (N=703), n (%)		*P* value (wave 1 vs all 3 waves)	
Age (years), mean (SD)		39.13 (10.84)	42.47 (12.01)	<.001		43.58 (12.14)		<.001	
**Age category (years)**					<.001				<.001
	18-24	26 (3.7)	38 (3.9)			24 (3.4)			
	25-34	246 (35.4)	256 (26)			156 (22.2)			
	35-44	238 (34.2)	306 (31.1)			225 (32)			
	45-54	107 (15.4)	202 (20.5)			154 (21.9)			
	55-65	63 (9.1)	136 (13.8)			107 (15.2)			
	Older than 65	15 (2.2)	45 (4.6)			37 (5.3)			
**Sex**					.10				.64
	Female	337 (48.5)	531 (54)			387 (55)			
	Male	353 (50.8)	445 (45.3)			312 (44.4)			
	Transgender	3 (0.4)	2 (0.2)			1 (0.1)			
	Do not identify as female, male, or transgender	2 (0.3)	5 (0.5)			3 (0.4)			
**Race**					.17				.17
	White	588 (84.6)	810 (82.4)			576 (81.9)			
	Black	59 (8.5)	73 (7.43)			47 (6.7)			
	Asian	28 (4)	56 (5.7)			47 (6.7)			
	Native Hawaiian or Pacific Islander	1 (0.1)	0 (0)			0 (0)			
	American Indian or Native Alaskan	0 (0)	0 (0)			0 (0)			
	Other	4 (0.6)	5 (0.51)			3 (0.4)			
	Multiracial	14 (2)	34 (3.46)			27 (3.8)			
**Ethnicity**					<.001				<.001
	Not Hispanic or Latin	552 (79.4)	901 (91.7)			662 (94.2)			
	Hispanic or Latino	143 (20.6)	82 (8.3)			41 (5.8)			
**Education**					<.001				<.001
	No high school diploma	1 (0.1)	3 (0.3)			3 (0.4)			
	High school graduate	33 (4.8)	98 (10)			78 (11.1)			
	Some college, no degree	85 (12.3)	209 (21.3)			152 (21.6)			
	Occupational or technical degree	15 (2.2)	33 (3.4)			25 (3.6)			
	Associate degree	41 (5.9)	106 (10.8)			91 (12.9)			
	Bachelor’s degree	402 (58)	365 (37.2)			228 (32.4)			
	Master’s degree	106 (15.3)	138 (14.1)			106 (15.1)			
	Professional school	8 (1.2)	16 (1.6)			11 (1.6)			
	Doctoral degree	2 (0.3)	12 (1.2)			9 (1.3)			
**Marital status**					<.001				.003
	Married or living with partner	541 (77.8)	579 (58.9)			391 (55.6)			
	Separated	11 (1.6)	10 (1)			7 (1)			
	Divorced	28 (4)	105 (10.7)			89 (12.7)			
	Widowed	3 (0.4)	11 (1.1)			7 (1)			
	Never married	112 (16.1)	278 (28.3)			209 (29.7)			
**Income (US $)**					<.001				.87
	Less than 10,000	22 (3.2)	48 (4.9)			35 (5)			
	10,000-19,999	58 (8.3)	80 (8.1)			59 (8.4)			
	20,000-29,999	89 (12.8)	113 (11.5)			82 (11.7)			
	30,000-39,999	67 (9.6)	130 (13.2)			99 (14.1)			
	40,000-49,999	111 (16)	112 (11.4)			84 (11.9)			
	50,000-59,999	121 (17.4)	111 (11.3)			75 (10.7)			
	60,000-79,999	89 (12.8)	142 (14.4)			99 (14.1)			
	80,000-99,999	72 (10.4)	107 (10.9)			74 (10.5)			
	100,000-199,999	63 (9.1)	121 (12.3)			82 (11.7)			
	200,000 or more	3 (0.4)	19 (1.9)			14 (2)			
**Employment**					<.001				.02
	Full-time	514 (74.1)	570 (58)			396 (56.3)			
	Part-time	56 (8.1)	123 (12.5)			97 (13.8)			
	Looking for work	26 (3.8)	55 (5.6)			37 (5.3)			
	Maternity leave	5 (0.7)	1 (0.1)			1 (0.1)			
	Not working due to health	21 (3)	48 (4.9)			35 (5)			
	Student	16 (2.3)	31 (3.2)			15 (2.1)			
	Retired	12 (1.7)	49 (5)			40 (5.7)			
	Keeping house	27 (3.9)	59 (6)			45 (6.4)			
	Other	17 (2.5)	47 (4.8)			37 (5.3)			

**Table 2:** Overall sample distribution and response rates by characteristic for those who responded in Wave 2 (at 3 months) and in Wave 3 (at 6 months).

Characteristics			Wave 1 (N=1678), n (%)		Wave 2 response rate of wave 1 participants (N=983), %		Wave 3 response rate of wave 1 participants (N=703), %		Wave 3 response rate of wave 2 participants (N=703), %
**Age category (years)**									
	18-24	64 (3.8)		59.4		37.5		63.2	
	25-34	502 (29.9)		51		31.1		60.9	
	35-44	544 (32.4)		56.3		41.4		73.5	
	45-54	309 (18.4)		65.4		49.8		76.2	
	55-65	199 (11.9)		68.3		53.8		78.7	
	Older than 65	60 (3.6)		75		61.7		82.2	
**Sex**									
	Female	868 (51.7)		61.2		44.6		72.9	
	Male	798 (47.6)		55.8		39.1		70.1	
	Transgender	5 (0.3)		40		20		50	
	Do not identify as female, male, or transgender	7 (0.4)		71.4		42.9		60	
**Race**									
	White	1398 (83.3)		57.9		41.2		71.1	
	Black	132 (7.9)		55.3		35.6		64.4	
	Asian	84 (5)		66.7		56		83.9	
	Native Hawaiian or Pacific Islander	1 (0.1)		0		0		0	
	American Indian or Native Alaskan	0 (0)		0		0		0	
	Other	9 (0.5)		55.6		33.3		60	
	Multiracial	48 (2.9)		70.8		56.3		79.4	
**Ethnicity**									
	Not Hispanic or Latin	1453 (86.6)		62		45.6		73.5	
	Hispanic or Latino	225 (13.4)		36.4		18.2		50	
**Education**									
	No high school diploma	4 (0.2)		75		75		100	
	High school graduate	131 (7.8)		74.8		59.5		79.6	
	Some college, no degree	294 (17.5)		71.1		51.7		72.7	
	Occupational or technical degree	48 (2.9)		68.8		52.1		75.8	
	Associate degree	147 (8.8)		72.1		61.9		85.8	
	Bachelor’s degree	767 (45.7)		47.6		29.7		62.5	
	Master’s degree	244 (14.5)		56.6		43.4		76.8	
	Professional school	24 (1.4)		66.7		45.8		68.8	
	Doctoral degree	14 (0.8)		85.7		64.3		75	
**Marital status**									
	Married or living with partner	1120 (66.7)		51.7		34.9		67.5	
	Separated	21 (1.3)		47.6		33.3		70	
	Divorced	133 (7.9)		78.9		66.9		84.8	
	Widowed	14 (0.8)		78.6		50		63.6	
	Never married	390 (23.2)		71.3		53.6		75.2	
**Income (US $)**									
	Less than 10,000	70 (4.2)		68.6		50		72.9	
	10,000-19,999	138 (8.2)		58		42.8		73.8	
	20,000-29,999	202 (12)		55.9		40.6		72.6	
	30,000-39,999	197 (11.7)		66		50.3		76.2	
	40,000-49,999	223 (13.3)		50.2		37.7		75	
	50,000-59,999	232 (13.8)		47.8		32.3		67.6	
	60,000-79,999	231 (13.8)		61.5		42.9		69.7	
	80,000-99,999	179 (10.7)		59.8		41.3		69.2	
	100,000-199,999	184 (11)		65.8		44.6		67.8	
	200,000 or more	22 (1.3)		86.4		63.6		73.7	
**Employment**									
	Full-time	1084 (64.6)		52.6		36.5		69.5	
	Part-time	179 (10.7)		68.7		54.2		78.9	
	Looking for work	81 (4.8)		67.9		45.7		67.3	
	Maternity leave	6 (0.4)		16.7		16.7		100	
	Not working due to health	69 (4.1)		69.6		50.7		72.9	
	Student	47 (2.8)		66		31.9		48.4	
	Retired	61 (3.6)		80.3		65.6		81.6	
	Keeping house	86 (5.1)		68.6		52.3		76.3	
	Other	64 (3.8)		73.4		57.8		78.7	

**Table 3:** Health conditions reported by those in the baseline (wave 1) sample who also responded in wave 2 (at 3 months) and in waves 2 and 3 (at both 3 and 6 months).

Condition	Responded in wave 1 only (N=695), mean (SD)	Responded in wave 1 and 2 only (N=983), mean (SD)	*P* value (wave 1 vs wave 1 and 2)	Responded in all 3 waves (N=703), mean (SD)	*P* value (wave 1 vs all 3 waves)
Hypertension	0.42 (0.49)	0.32 (0.47)	<.001	0.30 (0.46)	.005
High cholesterol	0.30 (0.46)	0.26 (0.44)	.08	0.27 (0.44)	.67
Heart disease	0.11 (0.32)	0.04 (0.19)	<.001	0.03 (0.17)	.03
Angina	0.11 (0.31)	0.03 (0.17)	<.001	0.02 (0.13)	.003
Heart attack	0.09 (0.29)	0.03 (0.17)	<.001	0.02 (0.14)	.006
Stroke	0.09 (0.28)	0.03 (0.17)	<.001	0.03 (0.16)	.12
Asthma	0.24 (0.42)	0.20 (0.40)	.06	0.20 (0.40)	.50
Cancer	0.07 (0.26)	0.07 (0.25)	.32	0.08 (0.27)	.007
Diabetes	0.24 (0.43)	0.12 (0.32)	<.001	0.10 (0.31)	.03
COPD^a^	0.11 (0.31)	0.05 (0.22)	<.001	0.05 (0.21)	.34
Arthritis	0.20 (0.40)	0.27 (0.44)	.004	0.28 (0.45)	.045
Anxiety	0.37 (0.48)	0.42 (0.49)	.03	0.41 (0.49)	.17
Depression	0.52 (0.50)	0.47 (0.50)	.04	0.45 (0.50)	.02
Allergies	0.44 (0.50)	0.52 (0.50)	.001	0.55 (0.50)	.005
Sciatica	0.31 (0.46)	0.25 (0.44)	.02	0.26 (0.44)	.09
Neck pain	0.52 (0.50)	0.40 (0.49)	<.001	0.38 (0.49)	.01
Trouble seeing	0.26 (0.44)	0.20 (0.40)	.007	0.20 (0.40)	.33
Dermatitis	0.18 (0.38)	0.16 (0.37)	.27	0.15 (0.36)	.25
Stomach trouble	0.36 (0.48)	0.32 (0.47)	.07	0.30 (0.46)	.02
Trouble hearing	0.15 (0.36)	0.09 (0.28)	<.001	0.08 (0.28)	.33
Trouble sleeping	0.50 (0.50)	0.54 (0.50)	.11	0.55 (0.50)	.22
Number of conditions	6.6 (3.67)	5.8 (2.96)	<.001	5.7 (2.88)	.08

^a^COPD: chronic obstructive pulmonary disease.

**Table 4:** Pain impact reported by those in the baseline (wave 1) sample who did not and did respond at wave 2 (at 3 months) and at waves 2 and 3 (at both 3 and 6 months).

Pain assessment	Responded in wave 1 only (N=695)	Responded in wave 1 and 2 only (N=983)	*P* value (wave 1 vs wave 1 and 2)	Responded in all 3 waves (N=703)	*P* value (wave 1 vs all 3 waves)
Nonspecific, proportion (SD)	0.80 (0.69)	0.55 (0.79)	<.001	0.64 (0.48)	<.001
Chronic, proportion (SD)	0.84 (0.37)	0.92 (0.26)	<.001	0.94 (0.24)	.016
Pain intensity, *z* score (SD)	0.85 (0.88)	0.62 (0.89)	<.001	0.50 (0.78)	.002
Pain interference, *z* score (SD)	0.80 (0.69)	0.55 (0.79)	<.001	0.57 (0.88)	.003
Impact Stratification Score (ISS), mean (SD)	22.07 (7.41)	19.34 (8.57)	<.001	18.99 (8.6)	.02
Oswestry Disability Index (ODI), mean (SD)	26.98 (15.99)	22.39 (15.99)	<.001	22.06 (16.09)	.15
Roland Morris Disability Questionnaire (RMDQ), mean (SD)	10.35 (6.63)	8.09 (6.46)	<.001	7.86 (6.42)	.04
Pain, Enjoyment of Life and General Activity scale (PEG), mean (SD)	4.33 (2.08)	3.74 (2.18)	<.001	3.58 (2.16)	<.001
Keele STarT Back Screening Tool (SBST), mean (SD)	4.10 (2.53)	3.48 (2.54)	<.001	3.38 (2.52)	.03

### Response Patterns in Wave 2

Table 5 presents the odds ratios (ORs) based on the logistic regression results after stepwise selection. The models identified the factors most related to responding in the wave 2 survey (Table 5; Model 1).

Respondents with longer response times (minutes) in wave 1 were less likely to respond to the wave 2 survey than those with longer response times in wave 1 (OR 0.98, 95% CI 0.97-0.99). Respondents who were Hispanic or Latino were also less likely to participate (compared with those who were not Hispanic or Latino, OR 0.69, 95% CI 0.49-0.96). In addition, those with a bachelor’s degree as their terminal degree (compared with all other education groups, OR 0.58) were less likely to respond in the following survey. Those with angina (OR 0.56), diabetes (OR 0.68), and sciatica (OR 0.72) compared with those without those conditions were less likely to respond to the wave 2 survey. Finally, those with greater pain intensity and interference (mean of the *z* scores for these measures; OR 0.75), and those with any specific back pain (OR 0.36-0.71) compared with those with nonspecific pain were less likely to respond to the following survey.

In contrast, older respondents (older than 45 years compared with 18-24 years old) were more likely to respond to the wave 2 survey (OR 2.63-3.79) and those whose marital status was divorced (OR 1.81) and separated (OR 1.77) were also more likely to respond the wave 2 survey. Those with allergies (OR 1.30) and trouble sleeping (OR 1.33) compared with those without those conditions were more likely to respond to the wave 2 survey.

**Table 5:** Results of models predicting response for wave 2 (3 months) and for waves 2 and 3 (3 and 6 months).

Variables					Model 1—Response for wave 2, odds ratio (95% CI)			Model 2—Response for waves 2 and 3, odds ratio (95% CI)
Time to complete (in minutes)					0.978^a^ (0.968-0.987)			0.978^a^ 0.968-0.987)
**Age (years)**								
	18-24 (reference)			reference			reference	
	25-34			1.340 (0.729-2.464)			0.931 (0.486-1.782)	
	35-44			1.731 (0.932-3.215)			1.573 (0.811-3.051)	
	45-54			2.633^b^ (1.371-5.056)			2.192^c^ (1.098-4.375)	
	55-65			2.868^b^ (1.434-5.736)			2.297^c^ (1.103-4.780)	
	Older than 65			3.785^b^ (1.559-9.189)			2.662^c^ (1.094-6.478)	
**Sex**								
	Male (reference)			reference			reference	
	Female			0.959 (0.763-1.206)			0.992 (0.786-1.252)	
	Transgender			0.264 (0.039-1.788)			0.199 (0.019-2.045)	
	Do not identify as female, male, or transgender			0.985 (0.166-5.842)			1.646 (0.289-9.376)	
**Race**								
	White (reference)			reference			reference	
	Black or African American			1.253 (0.826-1.901)			1.104 (0.715-1.706)	
	Asian			1.427 (0.846-2.405)			2.044^b^ (1.225-3.409)	
	Native American or Alaskan Native			4.305 (0.438-42.31)			1.625 (0.260-10.16)	
	Other			0.716 (0.179-2.864)			0.523 (0.115-2.372)	
	Multiracial			1.376 (0.691-2.739)			1.636 (0.851-3.143)	
**Ethnicity**								
	Hispanic or Latino			0.683^c^ (0.486-0.961)			0.540^b^ (0.362-0.806)	
**Education**								
	Terminal degree not bachelor’s degree (reference)			reference			reference	
	Bachelor’s degree			0.575^a^ (0.459-0.724)			0.446^a^ (0.346-0.575)	
**Marital status**								
	Neither divorced nor never married (reference)			reference			reference	
	Divorced			1.812^c^ (1.115-2.945)			1.894b (1.223-2.935)	
	Never married			1.769^a^ (1.318-2.375)			1.704^a^ (1.282-2.265)	
**Employment status**								
	Nonstudent (reference)			ns^d^ (reference)			reference	
	Student			ns (reference)			0.403^c^ (0.196-0.828)	
**Mean of pain intensity and pain interference** ***z*** **scores**					0.754^b^ (0.639-0.889)			0.707^a^ (0.595-0.841)
	**Type of pain**							
		Nonspecific pain (reference)	reference			reference		
		Specific and chronic pain	0.712^c^ (0.537-0.945)			0.624^b^ (0.477-0.816)		
		Specific and nonchronic pain	0.355^a^ (0.232-0.543)			0.330^a^ (0.209-0.520)		
	**Conditions**							
		Hypertension	ns (reference)			0.773^c^ (0.597-1.000)		
		Angina	0.559^c^ (0.333-0.939)			0.370^b^ (0.186-0.738)		
		Diabetes	0.678^c^ (0.495-0.929)			ns (reference)		
		Allergies	1.301^c^ (1.030-1.643)			1.321^c^ (1.048-1.665)		
		Sciatica	0.716^c^ (0.549-0.935)			ns (reference)		
		Trouble sleeping	1.330^c^ (1.052-1.682)			1.418^b^ (1.112-1.807)		

^a^*P*<.001.

^b^*P*<.01.

^c^*P*<.05.

^d^ns: nonsignificant in model.

### Response Patterns in Waves 2 and 3

We found that factors related to response in wave 2 were similar to those that related to response in waves 2 and 3 (Table 5; Model 2): time to complete, age, race, ethnicity, education, marital status, pain type, pain impact, and certain chronic conditions were associated with participation in both waves 2 and 3 of the survey.

In contrast to the model predicting response in the wave 2 survey (Model 1), those who identified as Asian (OR 2.04, 95% CI 1.23-3.41) were more likely to complete the waves 2 and 3 surveys than those who identified as White. Income was no longer significant, but being a student was associated with lower odds of responding (OR 0.40, 95% CI 0.20-0.83). Similarly, having diabetes or sciatica was no longer associated with response, but hypertension was associated with lower odds of responding (OR 0.77) compared with those without the condition.

### Description of Sample With Weighting by Nonresponse

Weights were created using the final stepwise logistic regression models shown in Table 5. To derive weights for responses in wave 2, the final model included age, sex, race, ethnicity, marital status (being divorced or never married), mean pain intensity and pain interference score, type of pain (specific and chronic pain or specific and nonchronic pain), and health conditions (angina, diabetes, allergies, sciatica, and trouble sleeping). To derive weights for response in waves 2 and 3, the final model included age, sex, race, ethnicity, marital status (being divorced or never married), employment (student or not student), mean pain intensity and pain interference score, type of pain (specific and chronic pain or specific and nonchronic pain), and health conditions (hypertension, angina, allergies, and trouble sleeping).

The inverse probability weighted samples of those completing wave 2 only and those completing waves 2 and 3 were similar demographically to the sample of all individuals who completed wave 1 for all categories except for income (Table S2 in Multimedia Appendix 1) [49]. Income was similar for the unweighted data and sample weights produced differences in the estimate of some income categories like the US $30,000-US $39,999 income range [50]. Estimated sample weights and weighted sample distributions did not vary between the backward elimination estimation–derived weights and those not using backward elimination estimation and were robust to model specification.

The greatest differences observed between the baseline and the weighted samples were in income, where the weighted sample had approximately US $5000 more in annual income than the baseline population. In addition, the wave 2 and 3 weighted samples had lower measured scores on the ISS, the ODI, the RMDQ, the PEG, and the SBST compared with the weighted participants in waves 2 only and the unweighted baseline sample (Table S3 and S4 in Multimedia Appendix 1). These differences were less than those between the unweighted samples (Table 4).

Respondents were generally older, less Hispanic, less likely to have a bachelor’s degree, less likely to be married, lower income, and less likely to be employed full-time when comparing unweighted and weighted wave 2 responses (Table S2 in Multimedia Appendix 1). Unweighted respondents had fewer health conditions and were less likely to have all health conditions except arthritis, anxiety, allergies, and trouble sleeping than weighted respondents in Wave 2 (Table S3 and S4 in Multimedia Appendix 1). The largest difference between unweighted and weighted respondents in wave 2 was in pain assessments. Unweighted respondents were much less likely to have nonspecific pain (0.55 vs 0.74), more likely to have chronic pain (0.92 vs 0.88) and have lower pain intensity and pain interference than weighted respondents. Unweighted respondents also scored systematically lower on the ISS, the ODI, the RMDQ, the PEG, and the SBST.

## Discussion

### Principal Results and Comparison With Previous Work

Comparisons of MTurk samples with the general US population have shown that MTurk participants tend to be younger, more educated, and less racially and ethnically diverse than the general population [11]. The characteristics of our baseline sample are consistent with this literature. This study extends the existing literature by describing nonresponse rates and predictors of nonresponse in a longitudinal study of MTurk participants with back pain, a population increasingly studied using survey methods to assess severity and changes over time [30,31].

We found that overall nonresponse was larger from wave 1 to wave 2 than between waves 2 and 3, even with a sample that was screened for higher quality respondents and those with lower quality responses were removed; however, it should be noted that since participants already responded in wave 2, there is selection bias as they have already participated in a follow-up survey wave. Previous research has shown that the majority of MTurk workers are frequent users of MTurk [51], participating in many tasks and using the platform regularly. Despite this, we found that even with regular outreach about the follow-up survey, response rates declined. While our survey focused on patients with back pain, our retention rate is on par with longitudinal studies that included more general samples of MTurk respondents [52], and greater than the average internet survey response rate of 40%-50% [53].

In addition, we found differential response rates by participant characteristics. Response rates were generally over 40% for all categories except those with relatively small sample sizes (ie, <2% of the overall sample). In this study, we have sample coverage for subgroups that reflect the larger population, indicating higher “positivity” as per Mercer et al [27]. Persistent respondents in our survey were older with higher income, less likely to be Hispanic, less educated, less likely to be employed full-time, and more likely to never have been married, all broadly consistent with previous nonresponse studies in MTurk [19]. In comparing response rates from wave 2 to wave 3, we also found less differential nonresponse than when comparing wave 2 with wave 1 and waves 2 and 3 with wave 1.

Participants who continued to respond in survey waves completed their previous surveys faster than those who did not respond and reported fewer health conditions. Our multivariate models largely supported these findings. Factors associated with increased response across survey waves were completing our previous survey faster, being divorced or never marrying, and having allergies or trouble sleeping, while factors negatively associated with response were being Hispanic or Latino, younger, having a bachelor’s degree, and having more reported pain intensity and interference. Our backward estimation specification allowed for individual categorical responses (ie, income, education, and employment) to be assessed independently, leading to instances where only specific subsets of a category are included in the model. The reference groups in these cases are any individuals not included in that category, sometimes making model interpretation unintuitive (ie, having an income of US $40,000-US $59,999). However, categories with the lowest response rates compared with wave 1 were included in the models, further supporting the model for the prediction of nonresponse. The effect of the inverse probability weights was robust to whether the subset of significant variables or all candidate variables were used to derive them. All of this mitigated “composition” issues that limit the ability to draw inferences as per Mercer et al [27].

The inverse probability weighted sample analysis produced sample demographics and condition prevalence rates that were similar between baseline respondents and those responding at subsequent waves except for pain assessments, which is most likely driven by changes in pain assessments over time rather than bias introduced by nonresponse. Average pain, according to different measures, was higher in the weighted sample than the unweighted samples, indicating that those with higher pain are more likely to be due to dropout and less likely to be due to a reduction in pain over time. Given the weighting was able to recreate wave 1 characteristic distributions even though the wave 2 and waves 2 and 3 samples were not similar to wave 1, we ensure that the composition of the weighted sample reflects our starting sample and would minimize inferential bias. These results were consistent with descriptive analysis of the survey waves, highlighting a potential issue with longitudinal analyses on those who report pain.

In each successive wave, those with more, nonchronic, and specific pain had a higher likelihood of nonresponse, potentially biasing analyses that do not account for differences in panel composition over time. Analyses using convenience panels need to account for changes in sample composition, particularly the loss of those with more severe and specific pain, and worse health. Estimates of impacts on pain may be biased if those with the most pain and the more specific pain are differentially dropping out of the panel. Hence, evaluating whether attrition impacts inferences about change in outcome measures such as pain is important.

Platforms like MTurk will continue to be used to collect survey data inexpensively and rapidly. Results from this nonresponse analysis should be considered in the context of general trends for MTurk workers. First, researchers should consider the underlying population that completes surveys on MTurk. Generally, the population who responds is younger, so higher nonresponse rates among younger individuals may be less concerning as that subgroup is already providing a higher proportion of responses than the general US population [13]. In addition, those with back pain tend to be older on average and come from predominantly non-White racial and ethnic groups [28]. However, high nonresponse rates among Hispanic or Latino participants may bias inferences as they represent smaller proportions of the base sample. In addition, analyses that account for specific populations like those with back pain should account for differences in sample composition over time, particularly around variables that may impact response rates and potential outcomes such as pain intensity or severity.

Regular outreach and follow-up have already been shown to increase response rates in longitudinal studies with MTurk. Inverse probability weighting can be used to correct for participant nonresponse. Given patterns of nonresponse, targeted surveys to populations that are either underrepresented or are more likely to drop out of surveys with MTurk can avoid issues with weighting small samples, especially when multiple successive survey waves exist.

### Limitations

This study had several limitations. First, since it focused on patients with back pain, its results should be interpreted with care and may not generalize to those without back pain. In addition, all data collection occurred during the COVID-19 pandemic. Response behavior may have differed during this time compared with before or after the pandemic. Finally, all data related to health conditions and pain assessments were self-reported. However, we applied various approaches to limit our sample to high-quality and truthful responses.

### Conclusions

Longitudinal studies on MTurk, particularly those exploring specific issues like the impacts of pain on health, should carefully consider how nonresponse will affect their study samples and whether samples drawn from these studies reflect the population of interest.

## Appendix

Multimedia Appendix 1 STROBE (Strengthening the Reporting of Observational Studies in Epidemiology) checklist and additional tables (weighted responses).

## Abbreviations

HIT: human intelligence task
ISS: Impact Stratification Score
MTurk: Amazon Mechanical Turk
ODI: Oswestry Disability Index
OR: odds ratio
PEG: Pain, Enjoyment of Life and General Activity scale
RMDQ: Roland Morris Disability Questionnaire
SBST: Keele STarT Back Screening Tool
STROBE: Strengthening the Reporting of Observational Studies in Epidemiology

## Data Availability

The dataset analyzed for the current study is not publicly available yet due to the project still being in progress, but the data are available from the first author upon reasonable request.
