# Supplementary material for: Dropout in a Longitudinal Survey of Amazon Mechanical Turk Workers With Low Back Pain: Observational Study
Source: Interact J Med Res. 2024 Nov 11;13:e58771. doi: 10.2196/58771 (PMC11589496; doi:10.2196/58771)
Supplement: Multimedia Appendix 1 [file ijmr_v13i1e58771_app1.docx]

**Table S1.** STROBE Statement—Checklist of items that should be included in reports of cohort studies.

|  | Item No | | Recommendation | Page No |
| --- | --- | --- | --- | --- |
| **Title and abstract** | 1 | | (*a*) Indicate the study’s design with a commonly used term in the title or the abstract | Title, Page 1 |
|  |  |  | (*b*) Provide in the abstract an informative and balanced summary of what was done and what was found | Page 1 |
| Introduction | | | | |
| Background/rationale | 2 | | Explain the scientific background and rationale for the investigation being reported | Pages 1-2 |
| Objectives | 3 | | State specific objectives, including any prespecified hypotheses | Pages 2-3 |
| Methods | | | | |
| Study design | 4 | | Present key elements of study design early in the paper | Title, Page 3 |
| Setting | 5 | | Describe the setting, locations, and relevant dates, including periods of recruitment, exposure, follow-up, and data collection | Page 3 |
| Participants | 6 | | (*a*) Give the eligibility criteria, and the sources and methods of selection of participants. Describe methods of follow-up | Pages 3 |
|  |  |  | (*b*) For matched studies, give matching criteria and number of exposed and unexposed | N/A |
| Variables | 7 | | Clearly define all outcomes, exposures, predictors, potential confounders, and effect modifiers. Give diagnostic criteria, if applicable | Pages 3-4 |
| Data sources/ measurement | 8 | | For each variable of interest, give sources of data and details of methods of assessment (measurement). Describe comparability of assessment methods if there is more than one group | Pages 3-4 |
| Bias | 9 | | Describe any efforts to address potential sources of bias | Page 4 |
| Study size | 10 | | Explain how the study size was arrived at | Page 3 |
| Quantitative variables | 11 | | Explain how quantitative variables were handled in the analyses. If applicable, describe which groupings were chosen and why | Pages 3-4 |
| Statistical methods | 12 | | (*a*) Describe all statistical methods, including those used to control for confounding | Page 4 |
|  |  |  | (*b*) Describe any methods used to examine subgroups and interactions | Page 4 |
|  |  |  | (*c*) Explain how missing data were addressed | N/A |
|  |  |  | (*d*) If applicable, explain how loss to follow-up was addressed | Page 4 |
|  |  |  | (*e*) Describe any sensitivity analyses | Page 4 |
| Results | | | |  |
| Participants | 13 | | (a) Report numbers of individuals at each stage of study—eg numbers potentially eligible, examined for eligibility, confirmed eligible, included in the study, completing follow-up, and analysed | Pages 4-9 |
|  |  |  | (b) Give reasons for non-participation at each stage | N/A |
|  |  |  | (c) Consider use of a flow diagram | Not included |
| Descriptive data | 14 | | (a) Give characteristics of study participants (eg demographic, clinical, social) and information on exposures and potential confounders | Pages 4-9, Tables 1 and 2 |
|  |  |  | (b) Indicate number of participants with missing data for each variable of interest | N/A |
|  |  |  | (c) Summarise follow-up time (eg, average and total amount) |  |
| Outcome data | 15 | | Report numbers of outcome events or summary measures over time | Pages 4-12, Tables 1, 2, 3, and 4 |
| Main results | 16 | | (a) Give unadjusted estimates and, if applicable, confounder-adjusted estimates and their precision (eg, 95% confidence interval). Make clear which confounders were adjusted for and why they were included | Pages 4-12, Table 5 |
|  |  | | (b) Report category boundaries when continuous variables were categorized | Page 4 |
|  |  | | (c) If relevant, consider translating estimates of relative risk into absolute risk for a meaningful time period | N/A |
| Other analyses | 17 | | Report other analyses done—eg analyses of subgroups and interactions, and sensitivity analyses | Page 12 |
| Discussion | | | | |
| Key results | 18 | Summarise key results with reference to study objectives | | Pages 12-14 |
| Limitations | 19 | Discuss limitations of the study, taking into account sources of potential bias or imprecision. Discuss both direction and magnitude of any potential bias | | Page 12-14 |
| Interpretation | 20 | Give a cautious overall interpretation of results considering objectives, limitations, multiplicity of analyses, results from similar studies, and other relevant evidence | | Pages 12-14 |
| Generalisability | 21 | Discuss the generalisability (external validity) of the study results | | Pages 12-14 |
| Other information | | | | |
| Funding | 22 | Give the source of funding and the role of the funders for the present study and, if applicable, for the original study on which the present article is based | | Page 14 |

**Table S2.** Characteristics of those participating in Wave 1 versus an inverse probability weighted sample of those who responded in Wave 2 (at 3 months) and in Waves 2 and 3 (at both 3 and 6 months).

|  | **Unweighted Wave 1 Participants** | **Unweighted Wave 1 and 2 only Participants** | **Weighted Responders in Wave 2** | | **Weighted Responders in Waves 2 and 3** |
| --- | --- | --- | --- | --- | --- |
|  | (N=1678) | (N=983) |  | |  |
| **Age, years (SD)** | 39.13 (10.84) | 42.47 (12.01) | 40.66 (11.37) | | 41.68 (11.78) |
| **Age category, N (%)** |  |  |  | |  |
| 18-24 | 64 (3.8%) | 38 (3.9%) | 3.9% | | 4.0% |
| 25-34 | 502 (29.9%) | 256 (26%) | 31.5% | | 27.5% |
| 35-44 | 544 (32.4%) | 306 (31.1%) | 33.7% | | 34.0% |
| 45-54 | 309 (18.4%) | 202 (20.5%) | 17.0% | | 18.4% |
| 55-65 | 199 (11.8%) | 136 (13.8%) | 10.7% | | 12.3% |
| 65+ | 60 (3.5%) | 45 (4.6%) | 3.2% | | 3.8% |
| **Gender, N (%)** |  |  |  | |  |
| Female | 868 (51.7%) | 531 (54%) | 51.3% | | 53.7% |
| Male | 798 (47.5%) | 445 (45.3%) | 47.8% | | 45.7% |
| Transgender | 5 (0.3%) | 2 (0.2%) | 0.4% | | 0.2% |
| Do not identify as female, male, or transgender | 7 (0.4%) | 5 (0.5%) | 0.4% | | 0.4% |
| **Race, N (%)** |  |  |  | |  |
| White | 1398 (83.3%) | 810 (82.4%) | 82.7% | | 82.1% |
| Black | 132 (7.8%) | 73 (7.4%) | 8.5% | | 8.0% |
| Asian | 84 (5.0%) | 56 (5.7%) | 4.9% | | 5.5% |
| Native Hawaiian/Pacific Islander | 1 (0.0%) | 0 (0%) | 0.0% | | 0.0% |
| Native American/Alaskan Native | 6 (0.3%) | 0 (0%) | 0.0% | | 0.0% |
| Other | 9 (0.5%) | 5 (0.5%) | 0.7% | | 0.5% |
| Multiracial | 48 (2.8%) | 34 (3.5%) | 3.0% | | 3.4% |
| **Ethnicity, N (%)** |  |  |  | |  |
| Not Hispanic or Latin | 1453 (86.5%) | 901 (91.7%) | 86.7% | | 91.0% |
| Hispanic or Latino | 225 (13.4%) | 82 (8.3%) | 13.3% | | 9.0% |
| **Education, N (%)** |  |  |  | |  |
| No high school diploma | 4 (0.2%) | 3 (0.3%) | 0.4% | | 0.6% |
| High school graduate | 131 (7.8%) | 98 (10%) | 8.4% | | 9.2% |
| Some college, no degree | 294 (17%) | 209 (21.3%) | 17.1% | | 20.9% |
| Occupational/technical degree | 48 (2.8%) | 33 (3.4%) | 3.0% | | 3.1% |
| Associate degree | 147 (8.7%) | 106 (10.8%) | 8.7% | | 10.5% |
| Bachelor's degree | 767 (45%) | 365 (37.1%) | 47.8% | | 39.8% |
| Master's degree | 244 (14%) | 138 (14%) | 12.8% | | 13.4% |
| Professional school | 24 (1.4%) | 16 (1.6%) | 1.1% | | 1.7% |
| Doctoral degree | 14 (0.8%) | 12 (1.2%) | 0.6% | | 0.8% |
| **Marital Status, N (%)** |  |  |  | |  |
| Married or living with partner | 1120 (66.7%) | 579 (58.9%) | 68.7% | | 62.1% |
| Separated | 21 (1.2%) | 10 (1%) | 1.0% | | 0.9% |
| Divorced | 133 (7.9%) | 105 (10.7%) | 6.4% | | 8.7% |
| Widowed | 14 (0.8%) | 11 (1.1%) | 1.0% | | 1.0% |
| Never married | 390 (23.2%) | 278 (28.3%) | 22.9% | | 27.3% |
| **Income, N (%)** |  |  |  |  | |
| Less than $10,000 | 70 (4.1%) | 48 (4.9%) | 8.4% | 7.3% | |
| $10,000 - $19,999 | 138 (8.2%) | 80 (8.1%) | 11.2% | 11.2% | |
| $20,000 - $29,999 | 202 (12.0%) | 113 (11.5%) | 10.6% | 11.2% | |
| $30,000 - $39,999 | 197 (11.7%) | 130 (13.2%) | 0.8% | 1.8% | |
| $40,000 - $49,999 | 223 (13.2%) | 112 (11.4%) | 11.7% | 14.5% | |
| $50,000 - $59,999 | 232 (13.8%) | 111 (11.3%) | 14.2% | 12.9% | |
| $60,000 - $79,999 | 231 (13.7%) | 142 (14.4%) | 13.9% | 11.2% | |
| $80,000 - $99,999 | 179 (10.6%) | 107 (10.9%) | 14.6% | 14.9% | |
| $100,000 - $199,999 | 184 (10.9%) | 121 (12.3%) | 10.8% | 10.6% | |
| $200,000 or more | 22 (1.3%) | 19 (1.9%) | 3.8% | 4.4% | |
| **Employment, N (%)** |  |  |  |  | |
| Full-time | 1084 (64.6%) | 570 (58%) | 62.7% | 60.2% | |
| Part-time | 179 (10.6%) | 123 (12.5%) | 11.5% | 11.2% | |
| Looking for work | 81 (4.8%) | 55 (5.6%) | 5.2% | 5.4% | |
| Maternity Leave | 6 (0.3%) | 1 (0.1%) | 0.1% | 0.1% | |
| Not working due to health | 69 (4.1%) | 48 (4.9%) | 4.1% | 4.8% | |
| Student | 47 (2.8%) | 31 (3.2%) | 2.6% | 3.0% | |
| Retired | 61 (3.6%) | 49 (5%) | 3.4% | 4.4% | |
| Keeping house | 86 (5.1%) | 59 (6%) | 5.8% | 6.5% | |
| Other | 64 (3.8%) | 47 (4.8%) | 4.5% | 4.5% | |

**Table S3.** Health conditions reported by those in the baseline (Wave 1) sample versus an inverse probability weighted sample of those who responded in Wave 2 (at 3 months) and in Waves 2 and 3 (at both 3 and 6 months).

| **Condition, mean (SD)** | **Unweighted Wave 1 Participants** | **Unweighted Wave 1 and 2 only Participants** | **Weighted Responders in Wave 2** | **Weighted Responders in Waves 2 and 3** |
| --- | --- | --- | --- | --- |
|  | (N=1678) | (N=983) |  |  |
| Hypertension | 0.36 (0.48) | 0.32 (0.47) | 0.37 | 0.36 |
| High cholesterol | 0.27 (0.44) | 0.26 (0.44) | 0.28 | 0.27 |
| Heart disease | 0.06 (0.25) | 0.04 (0.19) | 0.07 | 0.04 |
| Angina | 0.06 (0.23) | 0.03 (0.17) | 0.07 | 0.05 |
| Heart attack | 0.05 (0.23) | 0.03 (0.17) | 0.05 | 0.04 |
| Stroke | 0.05 (0.22) | 0.03 (0.17) | 0.05 | 0.05 |
| Asthma | 0.21 (0.41) | 0.20 (0.40) | 0.22 | 0.20 |
| Cancer | 0.06 (0.25) | 0.07 (0.25) | 0.05 | 0.05 |
| Diabetes | 0.16 (0.37) | 0.12 (0.32) | 0.16 | 0.15 |
| COPD | 0.07 (0.26) | 0.05 (0.22) | 0.06 | 0.06 |
| Arthritis | 0.24 (0.42) | 0.27 (0.44) | 0.24 | 0.25 |
| Anxiety | 0.39 (0.48) | 0.42 (0.49) | 0.41 | 0.41 |
| Depression | 0.48 (0.50) | 0.47 (0.50) | 0.48 | 0.49 |
| Allergies | 0.48 (0.50) | 0.52 (0.50) | 0.47 | 0.50 |
| Sciatica | 0.27 (0.44) | 0.25 (0.44) | 0.28 | 0.25 |
| Neck pain | 0.45 (0.49) | 0.40 (0.49) | 0.45 | 0.44 |
| Trouble seeing | 0.22 (0.41) | 0.20 (0.40) | 0.23 | 0.20 |
| Dermatitis | 0.16 (0.37) | 0.16 (0.37) | 0.16 | 0.14 |
| Stomach trouble | 0.33 (0.47) | 0.32 (0.47) | 0.34 | 0.32 |
| Trouble hearing | 0.11 (0.31) | 0.09 (0.28) | 0.11 | 0.08 |
| Trouble sleeping | 0.52 (0.49) | 0.54 (0.50) | 0.52 | 0.51 |

**Table S4.** Pain assessments reported by those in the baseline (Wave 1) sample versus an inverse probability weighted sample of those who responded in Wave 2 (at 3 months) and in Waves 2 and 3 (at both 3 and 6 months).

| **Pain assessment, mean (SD)** | **Unweighted Wave 1 Participants** | **Unweighted Wave 1 and 2 only Participants** | **Weighted Responders in Wave 2** | **Weighted Responders in Waves 2 and 3** |
| --- | --- | --- | --- | --- |
|  | (N=1678) | (N=983) |  |  |
| Nonspecific | 0.73 (0.44) | 0.55 (0.79) | 0.74 | 0.69 |
| Chronic | 0.88 (0.31) | 0.92 (0.26) | 0.88 | 0.91 |
| Pain intensity | 0.71 (0.89) | 0.62 (0.89) | 0.76 | 0.66 |
| Pain interference | 0.65 (0.75) | 0.55 (0.79) | 0.69 | 0.59 |
| Impact Stratification Score (ISS) | 20.4 (8.22) | 19.34 (8.57) | 20.7 | 19.7 |
| Oswestry Disability Index (ODI) | 24.29 (16.14) | 22.39 (15.99) | 24.4 | 22.8 |
| Roland Morris Disability Questionnaire (RMDQ) | 9.02 (6.62) | 8.09 (6.46) | 8.99 | 8.30 |
| Pain, Enjoyment of Life and General Activity scale (PEG) | 3.98 (2.15) | 3.74 (2.18) | 4.11 | 3.81 |
| Keele STarT Back Screening Tool (SBST) | 3.73 (2.55) | 3.48 (2.54) | 3.82 | 3.56 |
